# Editorial: Molecular and Cellular Plant Reproduction

**DOI:** 10.3389/fpls.2017.00199

**Published:** 2017-02-20

**Authors:** Dazhong Zhao, Kang Chong, Ravishankar Palanivelu

**Affiliations:** ^1^Department of Biological Sciences, University of Wisconsin-MilwaukeeMilwaukee, WI, USA; ^2^Key Laboratory of Plant Molecular Physiology, Institute of Botany, Chinese Academy of SciencesBeijing, China; ^3^School of Plant Sciences, University of ArizonaTucson, AZ, USA

**Keywords:** sexual and asexual reproduction, flowering time and flower development, embryo and fruit development, meiosis, sex determination, pollen tube growth, self-incompatibility and pollination, sterility and floral organ degeneration, epigenetics, gene regulatory networks and live-cell imaging

The world is now facing many severe problems due to increasing global demands for already limiting food and energy resources. Plant reproduction is crucial not only for producing offspring but also for improving crop quality and yield. Moreover, plant reproduction entails complex growth and developmental processes, which offer various opportunities for elucidating fundamental principles in biology. To provide a comprehensive update on the myriad of topics in the field of plant reproduction, we invited contributions for a Research Topic entitled “Molecular and Cellular Plant Reproduction”. After an overwhelming response, reflecting the active research being undertaken in plant reproduction, we published a total of 31 papers, including 1 hypothesis and theory, 4 perspectives, 12 reviews, and 14 original research articles. As a result, this special issue covers the broad scope and dynamics of studies on plant reproduction, ranging from a hypothesis of the sexual reproduction cycle, asexual reproduction, flowering time control, regulation of inflorescence architecture, floral stem cell termination, flower, lemma, palea, petal and anther development, anther dehiscence, sex determination, meiosis, pollen tube growth, self-incompatibility, pollination, embryogenesis, endosperm development, germline, antipodal and suspensor cell differentiation, fruit development, gynogenesis and androgenesis, to sterility caused by floral organ degeneration and abiotic stresses.

The life cycle of flowering plants features the alternation of diploid sporophyte and haploid gametophyte generations, in which a crucial step is to specify diploid reproductive cells from somatic cells. Animals generate diploid germlines during early embryonic development, while flowering plants determine reproductive cell lines post-embryonically. Bai proposes a sexual reproduction cycle (SRC) hypothesis, which generalizes sexual reproduction into an alternated three-step cycle consisting of meiosis, sex differentiation, and fertilization. In his hypothesis, Bai makes a remarkable attempt to address evolution as well as differences and similarities in sexual reproduction among various organisms, including fungi, plants, and animals. Sexual reproduction bestows selective advantages during evolution. Interestingly, Hojsgaard and Hörandl argue that “a little bit of sex” is helpful for apomictic plants to avoid genomic decay and extinction. Zhu et al. show that asexually propagated *Alternanthera philoxeroides* produces dysfunctional stamens due to decreased expression of B-class genes, suggesting that loss of sexual reproductive capacity might help evolution of asexual reproduction in clonal plants or *vice versa*.

Flower development is a long and complex process, which mainly includes four stages, i.e., vegetative to reproductive phase transition, specification of floral meristem identity, establishment of floral organ identity, and floral organ morphogenesis. In flowering plants, environmental and developmental cues trigger the conversion of the shoot apical meristem in adult plants to an inflorescence meristem, which subsequently gives rise to floral meristems. Reproductive cells that are specified from somatic cells in flowers initiate a germline lineage and then culminate with the production of gametes. Han et al. discuss recent findings about the role of cytokinin in inflorescence branching, which may contribute to the complexity of inflorescence architecture. Initiation and termination of floral stem cells in the right time and place are critical for flower formation. Sun and Ito review how transcriptional regulation and epigenetic machinery coordinately control floral stem cell termination during flower development. Using RNA-seq analysis, Zhang et al. identify ~24,000 flower-expressed genes at three stages, implying the need of complex gene networks for flower development and providing a valuable dataset for further studying genes important for flower development in *Arabidopsis* and other species. Flowers in monocots and dicots are structurally different in terms of their perianths. Lombardo and Yoshida discuss an interesting question about the origin of lemma and palea as well as regulation of their development by transcription factors in rice. Using *Gerbera hybrida*, which produces numerous petals, Li et al. profile differentially expressed genes in petals treated with gibberellin (GA) and abscisic acid (ABA), which provides a useful genomic resource for studying petal morphogenesis.

Flowering phase transition, floral meristem identity, and floral organ identity have been extensively studied. However, what is less clear and what has received less attention is how floral organs are established, especially the formation of anther and ovule, where male (pollen) and female gametophytes are differentiated from somatic cells, respectively. The anther includes reproductive microsporocytes (male meiocytes) and somatic anther wall cells. Kelliher et al. compare anther cell differentiation among *Arabidopsis*, rice, and maize and also discuss potential functions of small RNAs in anther patterning. Atallah and Banks review recent genetic and biochemical studies on how the pheromone antheridiogen (gibberellins) determines the sex of gametophytes in ferns via gibberellin signaling and biosynthetic pathways. Moreover, transcriptome analysis reveals differentially expressed genes in male and female flowers of the monoecious species *Quercus suber*, which may shed light on gene networks regulating sex specification Rocheta et al. Wang et al. show that the miR319a-targeted *TCP24* controls anther dehiscence by negatively regulating secondary cell wall thickening of the endothecium.

Meiosis is an essential process for sexual reproduction, leading to the formation of male and female gametophytes. Li et al. report their development of MeioBase, which provides a valuable database and various tools for meiosis research. Wu et al. show that the meiosis-specific cyclin-like protein SDS (SOLO DANCERS) is required for double-strand break (DSB) formation in rice, rather than DSB repair found in *Arabidopsis*, revealing distinct functions of this conserved protein in plants. Qu et al. highlight the role of the actin cytoskeleton in pollen tube growth from several different angles, including the spatial distribution of actin filaments in the pollen tube, use of actin markers for dissecting organization and dynamics of actin filaments in growing pollen tubes, actin filament dynamics in the pollen tube, as well as functions and mechanisms of actin-binding proteins (ABPs) in actin filament generation and dynamics. McDowell et al. show that disruption of *A**MINOPHOSPHO**L**IPID* *A**TPASE 6* (*ALA6*) and *ALA7* genes causes changes in the properties of the pollen tube plasma membrane, demonstrating a role of *ALA* genes in pollen fitness under normal and temperature-stress conditions. In addition, Vogler et al. describe a live-cell imaging method for characterizing distinctive vesicle and F-actin polarization during pollen activation, germination, and the establishment of pollen tube tip growth in *Arabidopsis*.

Williams et al. review the molecular mechanism underlying S-RNase-based self-incompatibility by discussing uptake of S-RNase into pollen tubes, formation of SLF (S-locus F-box)-containing SCF complexes, the nature of SLF proteins, and the fate of non-self S-RNases in pollen tube. Brito et al. found that pollination promotes female gametophyte development in immature tobacco flowers; however, the nature of these induction signals and the sensing mechanisms in the female gametophyte remain mysterious.

Five reviews and 3 research papers cover embryogenesis, highlighting the importance of parent-of-origin genes, epigenetic control, and hormone signaling during embryo development. Luo et al. discuss the requirement of gamete-delivered transcripts and imprinted genes for zygotic division and cell fate determination during embryogenesis. They also briefly discuss the regulation of imprinted genes in embryos. In particular, Russell and Jones anticipate more complex functions of male gamete-expressed genes during embryogenesis after discussing initiation and origin of the male germ lineage in flowering plants, features of sperm cells, expression of male germ-derived transcripts, common but divergent profiles of transcriptomes and proteomes in egg and sperm, transposable elements in pollen and male germline, contribution of sperm cells to fertilization, as well as male expression and evolutionary selection.

Epigenetic control is critical for plant growth and development, including sexual reproduction. Shi et al. review how DNA methylation, histone methylation, polycomb silencing, histone acetylation, as well as small and long non-coding RNAs are involved in flowering time regulation, floral organogenesis, meiosis, embryogenesis, and the endosperm development in rice. Using the whole genome analysis, Jung et al. detected repression-related epigenetic histone modifications in a large number of germline genes, suggesting that epigenetic control is a main mechanism for repressing germline genes during sexual reproduction. Tonosaki and Kinoshita summarize the roles of various polycomb repressive complex 2 (PRC2) proteins in the cereal endosperm development and suggest that monocots and eudicots may use different programs to control endosperm development. Moreover, Köhler and Lafon-Placette propose that the active PRC2 in the central cell and endosperm contributes to the transposon silencing in the egg cell and embryo by maintaining DNA hypomethylation activities.

Localization and mutant studies on PIN and auxin signaling discover a correlation between auxin signaling and antipodal cell proliferation in maize, which may provide a clue about the function of antipodal cells in embryo development Chettoor and Evans; this is particularly important given that the function of antipodal cells has not been assigned even though their presence in angiosperms is near universal and has been known for a long time. Scarlet runner bean (SRB) has been used as a model for studying embryo development for over 40 years due to its giant embryo. Henry and Goldberg review recent progress in identifying gene regulatory networks controlling suspensor differentiation and development using comparative genomic tools. Su et al. show that auxin and cytokinin signaling are engaged in establishment of the shoot-root axis during somatic embryogenesis in *Arabidopsis*, which provides insight into apical-basal patterning in embryo development.

As the final product of reproduction, fruit is vitally important for seed development in plants and serves as an indispensable source of food for animals, including humans. Wang et al. review progress on dissecting developmental and genetic mechanisms regarding the evolution and development of fruit size, shape, color, and morphological novelties in *Solanaceae*. Portemer et al. report that mutants in gynogenesis or androgenesis cannot be readily found in mutant screens; they demonstrate this outcome by screening for mutants in an EMS-mutagenized *Arabidopsis* population. Flower development is highly sensitive to environmental factors. Floral organ degeneration or abortion occurs naturally, leading to unisexual or fully sterile flowers. Abiotic stresses cause abnormal flower development and consequently affect crop yield and quality. Smith and Zhao review physiological and molecular genetic mechanisms underlying floral organ degeneration and fertility. In addition, they discuss how abiotic stresses, including heat, cold, and drought, affect sexual reproduction at morphological and genetic levels. Further understanding of sterility caused by floral organ degeneration and abiotic stresses will help plant scientists use both traditional and molecular breeding approaches to develop crops with high yield in stress environments.

This special research topic covers a variety of important issues, providing evidence of the explosive interest and considerable progress that has been made recently in plant reproduction. We envision that the articles compiled in this E-book will draw attention of all plant biologists to this research topic and help solve challenging and unresolved questions. Employment of combinatorial approaches involving molecular genetic tools and emerging technologies, such as fluorescence-based imaging techniques and next generation sequencing, will advance future studies on all reproductive processes in model plants, crops, and trees.

Finally, we would like to thank all the authors, reviewers, and the Frontiers editorial office for their contributions and hard work that made this interesting and special issue a reality.

## Author contributions

DZ wrote the editorial. KC and RP revised it.

### Conflict of interest statement

The authors declare that the research was conducted in the absence of any commercial or financial relationships that could be construed as a potential conflict of interest.

